# Two new genera and eight new species of jumping spiders (Araneae, Salticidae) from Xishuangbanna, Yunnan, China

**DOI:** 10.3897/zookeys.952.51849

**Published:** 2020-07-23

**Authors:** Yejie Lin, Shuqiang Li

**Affiliations:** 1 Hebei Key Laboratory of Animal Diversity, College of Life Science, Langfang Normal University, Langfang 065000, China Langfang Normal University Langfang China; 2 Institute of Zoology, Chinese Academy of Sciences, Beijing 100101, China Institute of Zoology, Chinese Academy of Sciences Beijing China

**Keywords:** All Species Inventory, taxonomy, tropical rainforest, XTBG

## Abstract

Two new genera and eight new species of jumping spiders from Xishuangbanna Tropical Botanical Garden (XTBG) are diagnosed, described, and illustrated. The new genera are *Dendroicius***gen. nov.** (type species *D. hotaruae***sp. nov.** (♂♀)) and *Megaeupoa***gen. nov.** (type species *M. yanfengi***sp. nov.** (♂♀)). The new species are *Colyttus yiwui***sp. nov.** (♂♀), *Euophrys xuyei***sp. nov.** (♂♀), *Foliabitus weihangi***sp. nov.** (♂♀), *Nigorella mengla***sp. nov.** (♂♀), *Onomastus chenae***sp. nov.** (♂♀), and *Synagelides platnicki***sp. nov.** (♂♀). A new combination is proposed: *Megaeupoa gravelyi* (Caleb, 2018), **comb. nov.**, ex *Brettus* Thorell, 1895. Two new synonyms have been proposed: *Irura prima* (Żabka, 1985), **syn. nov.** with *Irura mandarina* Simon, 1903; *Evarcha digitata* Peng & Li, 2002, **syn. nov.** with *Ptocasius montiformis* Song, 1991.

## Introduction

Salticidae Blackwall, 1841, or jumping spiders, is the largest spider family, with 6183 species in 646 genera worldwide (WSC 2020). Of the 5078 species of spiders described from China, 526 are jumping spiders (Li 2020a). This paper describes two new genera and eight new species of jumping spiders from Xishuangbanna Tropical Botanical Garden (XTBG), Yunnan, southwestern China.

Xishuangbanna Tropical Botanical Garden is located on Hulu Island in Menglun Township, Mengla County. XTBG is separated from the mainland by the Luosuo River, a tributary of the Mekong River (known as the Lancang River in China). XTBG’s 11.25 square kilometers includes a 2.50 square kilometer patch of well-preserved primary tropical rainforest, the main research area of our “All Species Inventory” on XTBG spiders for the past 20 years.

Until now, the Xishuangbanna spider checklist included 782 species in 46 families (Li 2020a). The species diversity from XTBG is greater than the number of species found in thoroughly studied regions, such as the United Kingdom, Norway, and Denmark (Nentwig et al. 2020). From our long-term study, we expect to find more spider species from XTBG.

## Materials and methods

Specimens were collected by fogging in XTBG. All specimens were preserved in 100% ethanol. Epigynes were cleared in trypsin enzyme solution to dissolve non-chitinous tissues. Specimens were examined under a LEICA M205C stereomicroscope. Photomicroscope images were taken with an Olympus C7070 zoom digital camera (7.1 megapixels). Photos were stacked with Helicon Focus (version 6.7.1) or Zerene Stacker (version 1.04) and processed in Adobe Photoshop CC 2018. All measurements are in millimeters. Eye sizes are measured as the maximum diameter from either the dorsal or frontal view. Leg measurements are given as follows: total length (femur, patella+tibia, metatarsus, tarsus); however, in *Synagelides platnicki* sp. nov., because of the long patella, we use (femur, patella, tibia, metatarsus, tarsus). All specimens are deposited in the Institute of Zoology, Chinese Academy of Sciences (IZCAS) in Beijing, China.

Abbreviations used in the text and figures:

**AER** anterior eye row

**AERW** anterior eye row width

**AG** accessory gland

**AL** abdomen length

**ALE** anterior lateral eye

**AME** anterior median eye

**AW** abdomen width

**BH** basal hematodocha

**C** conductor

**CD** copulatory duct

**CO** copulatory opening

**Cy** cymbium

**dEA** dorsal embolic apophysis

**DH** distal hematodocha

**DTA** dorsal tibial apophysis

**E** embolus

**EFL** eye field length

**EO** embolic opening

**ED** embolic disc

**EP** embolic part

**ES** embolic sheath

**FD** fertilization duct

**iTA** inferior terminal apophysis

**LE** lamella of embolus

**lTA** lateral terminal apophysis

**M** membrane

**MA** median apophysis

**mDTA** mesal branch of DTA

**MH** median hematodocha

**MS** median septum

**MTP** membranous tegular peak

**PA** patellar apophysis

**PER** posterior eye row

**PERW** posterior eye row width

**PLE** posterior lateral eye

**PME** posterior median eye

**PS** primary spermathecae

**PTA** posterior terminal apophysis

**RSDL** retrolateral sperm duct loop

**RTA** retrolateral tibial apophysis

**rMA** retrolateral median apophysis

**S** spermathecae

**SP** spur on mesal branch of conductor

**Sp** spine

**SD** sperm duct

**SS** secondary spermathecae

**ST** subtegulum

**T** tegulum

**TA** terminal apophysis

**TL** tegular lobe

**TS** tegular sclerite

**VTB** ventral tibial bump

**W** window

## Taxonomy

### Family Salticidae Blackwall, 1841

#### Genus Colyttus

Taxon classificationAnimaliaAraneaeSalticidae

Thorell, 1891

49BBC5A0-B1E3-5D79-82FB-2F8F5AD7D267

##### Type species.

*Colyttus bilineatus* Thorell, 1891.

#### Colyttus yiwui sp. nov.

Taxon classificationAnimaliaAraneaeSalticidae

8CECB201-CE0C-5225-83C3-8E1F416C0A1B

http://zoobank.org/022B0848-4F66-4B45-83D1-17E9AFDD2316

Figures 1 , 2

##### Type material.

***Holotype*** ♂(IZCAS-Ar40379), China: Yunnan: Xishuangbanna, Mengla County, Menglun Township, XTBG, Leprosy Village, 21.8932N, 101.2883E, elevation ca 550 m, 27.IX.2017, Zhigang Chen leg. ***Paratypes*** 7♂3♀(IZCAS-Ar40380–Ar40389), same data as holotype.

**Figure 1.**
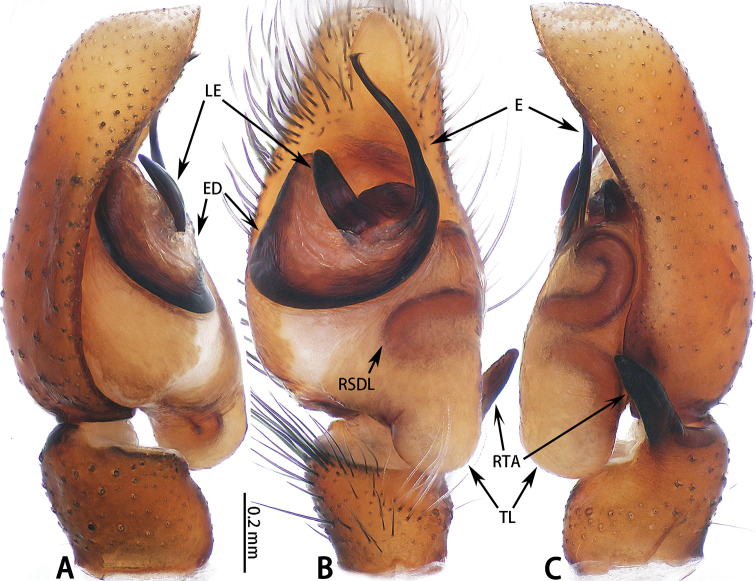
Palp of *Colyttus yiwui* sp. nov., male holotype. **A** prolateral **B** ventral **C** retrolateral.

##### Etymology.

This species is named after Mr. Yiwu Zhu, who has helped us greatly with this research; noun (name) in genitive case.

##### Diagnosis.

The males of *Colyttus yiwui* sp. nov. are similar to *C. proszynskii* Caleb, Chatterjee, Tyagi, Kundu & Kumar, 2018 by having a similarly-shaped embolus. However, *C. zhui* sp. nov. can be distinguished by the well-developed tegular lobe (vs. less well-developed in *C. proszynskii*), the ratio of the length of the embolus to the width of the embolic disc 1:1 (vs. 2:1 in *C. proszynskii*), the curved embolic tip (vs. straight in *C. proszynskii*) and the blunt lamella of the embolus (vs. pointed in *C. proszynskii*).

##### Description.

**Figure 2.**
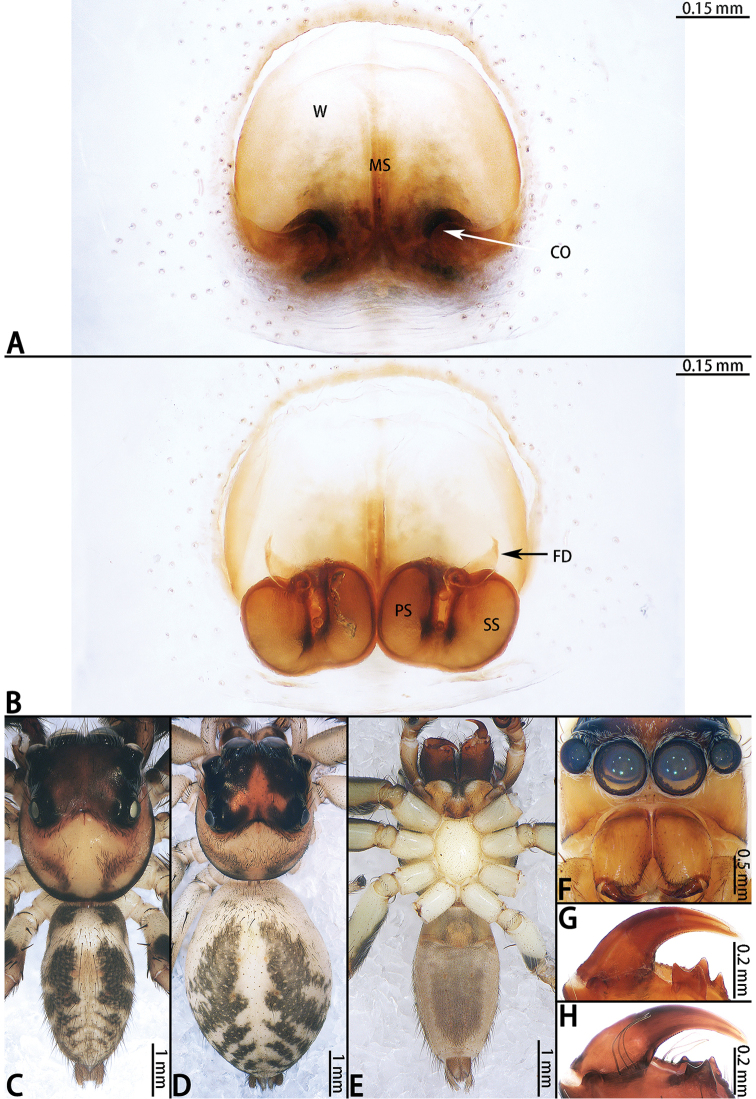
*Colyttus yiwui* sp. nov., female paratype and male holotype. **A** epigyne, ventral **B** vulva, dorsal **C** male holotype habitus, dorsal **D** female paratype habitus, dorsal **E** holotype habitus, ventral **F** frontal view of female paratype. **G** dorsal view of chelicerae, male holotype **H** dorsal view of chelicerae, female paratype.

**Male** (Figs 1, 2C, E, G). Total length 6.76. Carapace 3.36 long, 2.72 wide. Abdomen 3.40 long, 1.85 wide. Clypeus 0.05 high. Eye sizes and inter-distances: AME 0.67, ALE 0.45, PLE 0.43, AERW 2.21, PERW 2.12, EFL 1.44. Legs: I 6.93 (2.45 + 2.66 + 1.22 + 0.60), II 5.55 (1.63 + 2.15 + 1.22 + 0.55), III 5.81 (1.80 + 1.95 + 1.46 + 0.60), IV 6.04 (1.90 + 2.05 + 1.49 + 0.60). Carapace yellow-brown with black edge, eye region dark brown, with black rings around eyes. Fovea longitudinal, situated between PLEs. Clypeus black, covered with white setae. Chelicerae dark brown with two promarginal teeth and one retromarginal fissident tooth with two cusps. Endites, labium and sternum brown. Leg I black, other legs pale yellow except femora with black pattern. Abdomen elongated oval, dorsum with two pairs of muscle depressions medially, irregular yellow stripe across entire surface and bifurcated posteriorly, covered with brown setae and sparse, long setae; venter brown. Spinnerets brown.

Palp (Fig. 1A–C): Tibia stocky, slightly wider than long, with relatively long RTA; cymbium longer than wide; bulb approximately as long as wide; lamella of embolus blunt, embolus long, connected to embolic disc, embolic tip curved.

**Female** (Fig. 2A, B, D, F, H). Total length 4.82. Carapace 2.04 long, 1.63 wide. Abdomen 2.78 long, 1.61 wide. Clypeus 0.12 high. Eye sizes and inter-distances: AME 0.47, ALE 0.28, PLE 0.27, AERW 2.38, PERW 2.28, EFL 1.69. Legs: I 6.16 (1.96 + 2.43 + 1.16 + 0.61), II 5.00 (1.53 + 1.96 + 1.01 + 0.50), III 5.10 (1.59 + 1.66 + 1.31 + 0.54), IV 5.71 (1.66 + 1.98 + 1.53 + 0.54). Habitus similar to that of male except paler.

Epigyne (Fig. 2A, B) as long as wide, windows large, separated by median septum; copulatory openings on each side of septum located posteriorly; copulatory ducts indistinct, primary spermathecae smaller than secondary spermathecae, overall U-shaped; fertilization ducts originating from the anterior entolatetal edge of secondary spermathecae, extending almost transversely.

##### Distribution.

Known only from the type locality in Yunnan, China.

#### Genus Dendroicius gen. nov.

Taxon classificationAnimaliaAraneaeSalticidae

B5017932-BF6A-581C-8E17-BF77314609DA

http://zoobank.org/91408531-896D-4729-8C90-AFA460B25E09

##### Type species.

*Dendroicius hotaruae* sp. nov.

##### Etymology.

The generic name is a combination of the word “*Dendro*”, referring to the habitat of the genus, and the generic name *Icius* Simon, 1876. The gender is masculine.

##### Diagnosis.

*Dendroicius* gen. nov. can be easily distinguished from *Icius* by the following characters: the male without a stridulatory apparatus; palpal tibia with a dorsal apophysis, dorsal embolic apophysis of bulb near the tegular sclerite; epigyne with a large hood posteriorly, posterior to copulatory opening, copulatory opening circular, depression around copulatory opening, spermathecae posterior to copulatory opening, copulatory ducts curved, fertilization ducts folded 90°, well-developed.

##### Composition.

The new genus currently includes only one species: *Dendroicius hotaruae* sp. nov.

#### Dendroicius hotaruae sp. nov.

Taxon classificationAnimaliaAraneaeSalticidae

B490339D-AA0F-5AAE-B24B-46011D893D4B

http://zoobank.org/72556AE0-B460-402A-99B9-BA4795328B5E

Figures 3 , 4

##### Type material.

***Holotype*** ♂(IZCAS-Ar40390), China: Yunnan: Xishuangbanna, Mengla County, Menglun Township, XTBG, Leprosy Village, 21.8986N, 101.2683E, elevation ca 550 m, 27.IX.2017, Zilong Bai leg. ***Paratypes*** 1♂4♀(IZCAS-Ar40391–Ar40395), same data as holotype.

##### Etymology.

The species is named after Ms. Hotaru Amamiya, who helped us greatly with this research; noun (name) in genitive case.

##### Diagnosis.

Same as for the genus.

##### Description.

**Figure 3.**
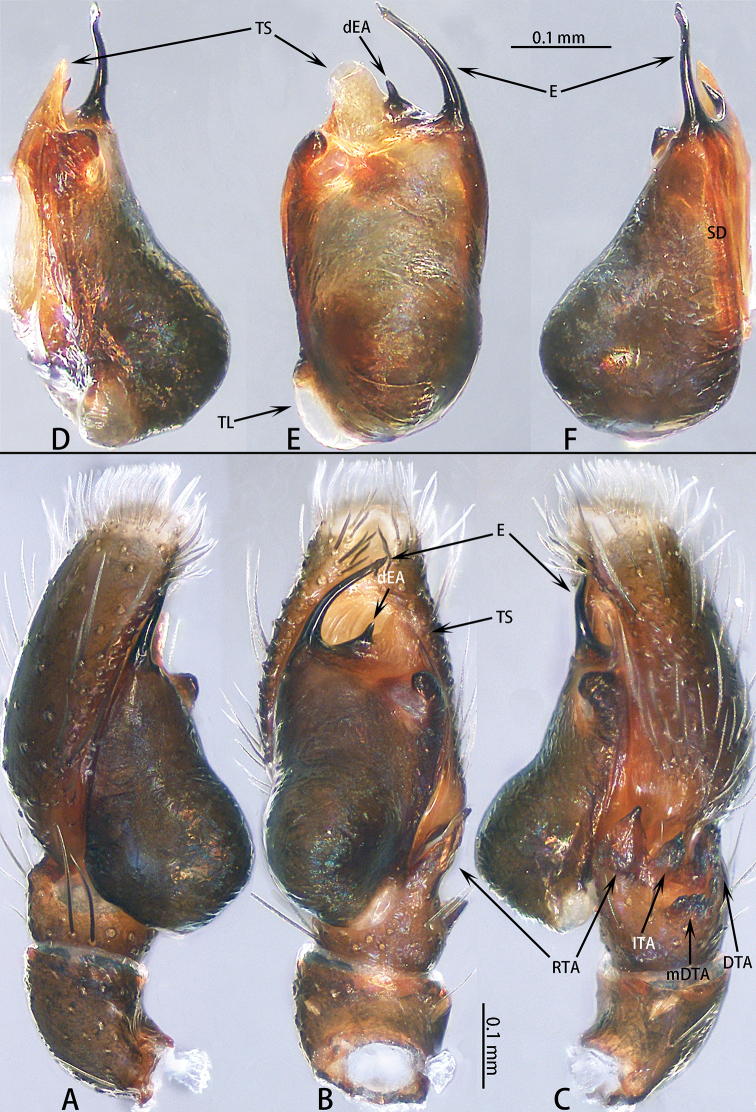
Male palp of *Dendroicius hotaruae* sp. nov. **A–C** holotype, left palp; **D, E** paratype, right palp. **A** prolateral **B** ventral **C** retrolateral **D** bulb, retrolateral **E** same, ventral **F** same prolateral.

**Figure 4.**
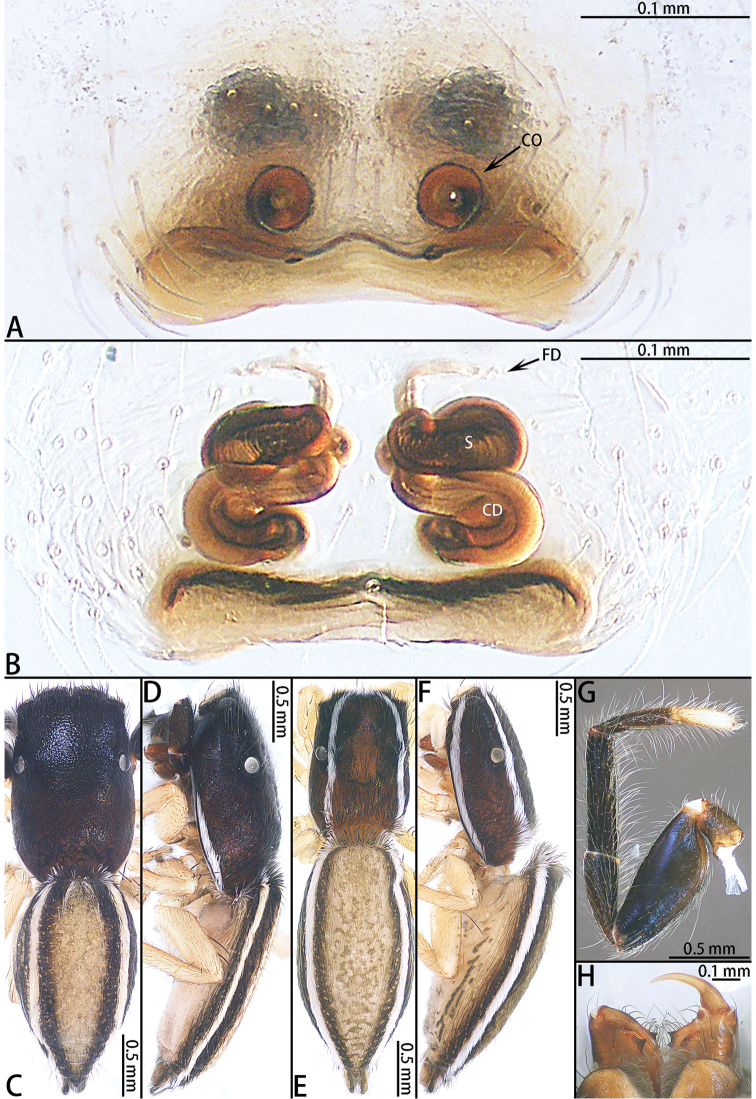
*Dendroicius hotaruae* sp. nov., female paratype and male holotype. **A** epigyne, ventral **B** vulva, dorsal **C** male holotype habitus, dorsal **D** same, lateral **E** female paratype habitus, dorsal **F** same, lateral **G** prolateral view of male left leg I **H** ventral view of female chelicerae.

**Male** (Figs 3, 4C, D, G). Total length 3.24. Carapace 1.58 long, 1.03 wide. Abdomen 1.88 long, 1.03 wide. Clypeus 0.03 high. Eye sizes and inter-distances: AME 0.32, ALE 0.16, PLE 0.16, AERW 0.85, PERW 0.94, EFL 0.56. Legs: I 2.54 (0.82 + 1.09 + 0.33 + 0.30), II 1.89 (0.59 + 0.71 + 0.36 + 0.23), III 1.86 (0.57 + 0.61 + 0.41 + 0.27), IV 2.46 (0.77 + 0.90 + 0.48 + 0.31). Carapace dark brown, darker in eye field, almost square, covered with black setae, edge with white setal stripe originating medially, thoracic part sloping acutely. Fovea indistinct. Clypeus black, anterior margin with long setae. Chelicerae black, with one retromarginal fissident tooth with two cusps and one retromarginal tooth. Endites brown. Labium brown. Sternum colored as endites, covered with sparse setae. Leg I black, others yellow. Abdomen elongated oval, dorsum pale brown with one pair of stripes of dense white setae, darker around stripes; venter pale yellow.

Palp (Fig. 3A–F) patella dark brown, slightly wider than long, covered with setae; tibia slightly wider than long, retrolateral tibial apophysis slightly longer than wide, lateral terminal apophysis darker, serrated along edge, dorsal tibial apophysis with small, serrated mesal branch; cymbium longer than wide, slightly longer than the length of the bulb in retrolateral view; bulb longer than wide, with sperm duct extending along margin; embolus short, half as long as bulb, needle shaped; dorsal embolic apophysis small, one fifth the length of embolus; tegular sclerite sheet-like, adjacent to dorsal embolic apophysis.

**Female** (Fig. 4A, B, E, F, H). Total length 3.52. Carapace 1.38 long, 0.90 wide. Abdomen 2.13 long, 1.09 wide. Clypeus 0.05 high. Eye sizes and inter-distances: AME 0.28, ALE 0.16, PLE 0.09, AERW 0.79, PERW 0.88, EFL 0.62. Legs: I 1.63 (0.55 + 0.62 + 0.26 + 0.20), II 1.40 (0.46 + 0.52 + 0.24 + 0.18), III 1.55 (0.49 + 0.48 + 0.30 + 0.28), IV 2.13 (0.66 + 0.75 + 0.39 + 0.33). Appearance of abdomen and legs as in male but carapace with two pairs of white setal latero-marginal stripes from lateral sides of AME and two longitudinal stripes of white setae from AMEs along PLE to the rear margin of carapace. All legs yellow and abdomen laterally with black pattern.

Epigyne (Fig. 4A, B) wider than long, with a wide hood posteriorly; copulatory openings circular; copulatory ducts long, curved medially; accessory gland indistinct; spermathecae oval; fertilization ducts folded 90°, well-developed.

##### Distribution.

Known only from the type locality in Yunnan, China.

#### Genus Euophrys

Taxon classificationAnimaliaAraneaeSalticidae

C. L. Koch, 1834

9FC8041E-FE16-5DAC-8618-9FD4AF048528

##### Type species.

*Aranea frontalis* Walckenaer, 1802.

#### Euophrys xuyei sp. nov.

Taxon classificationAnimaliaAraneaeSalticidae

DBD1121F-D25F-5B7E-A8CE-BBEFFA36770A

http://zoobank.org/892D8E78-05A9-443D-A6A4-E85091DF1D33

Figures 5 , 6

##### Type material.

***Holotype*** ♂(IZCAS-Ar40396), China: Yunnan: Xishuangbanna, Mengla County, Menglun Township, XTBG, Leprosy Village, 21.8932N, 101.2883E, elevation ca 550 m, 21.IX.2017, Zhigang Chen leg. ***Paratypes*** 1♂1♀(IZCAS-Ar40397, Ar40398), same data as holotype.

##### Etymology.

The species is named after Mr. Ye Xu, who helped us greatly with this research; noun (name) in genitive case.

##### Diagnosis.

*Euophrys xuyei* sp. nov. can be easily distinguished from other species by the following characters: male palpal tibia slightly longer than RTA in retrolateral view, tapering to a slightly hooded tip; bulb with tegular lobe covering tibia; embolic terminus flat, with small cusps; epigyne with copulatory openings on each side of median septum located posteriorly; copulatory ducts around spermathecae; accessory glands adjacent to copulatory openings.

##### Description.

**Figure 5.**
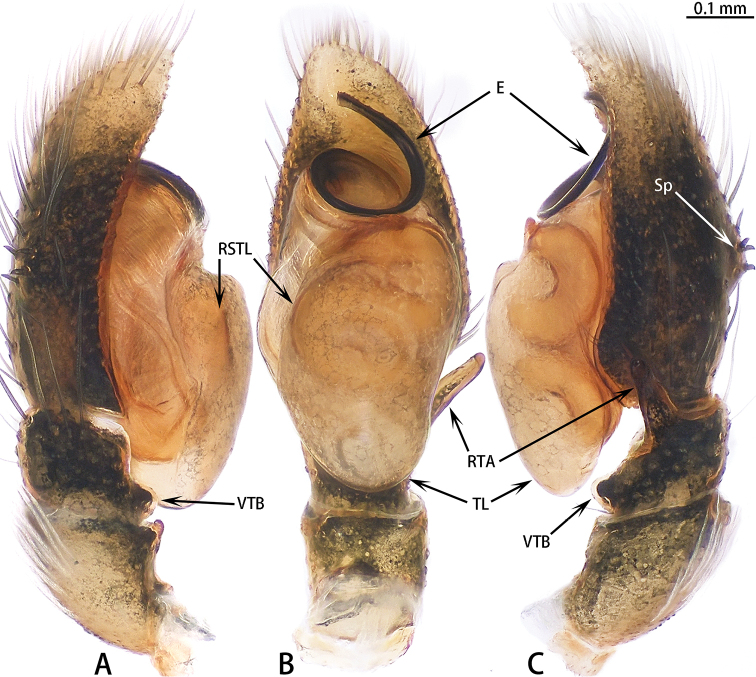
Palp of *Euophrys xuyei* sp. nov., male holotype. **A** prolateral **B** ventral **C** retrolateral.

**Figure 6.**
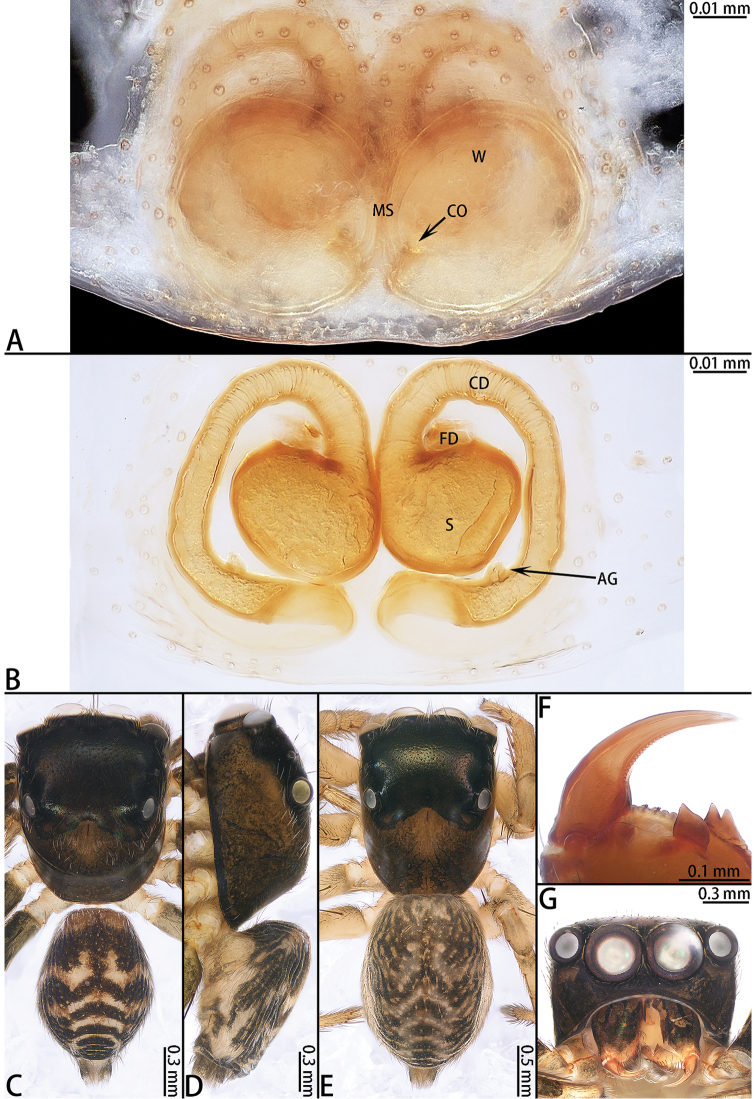
*Euophrys xuyei* sp. nov., female paratype and male holotype. **A** epigyne, ventral **B** vulva, dorsal **C** male holotype habitus, dorsal **D** same, lateral **E** female paratype habitus, dorsal **F** dorsal view of chelicerae, male paratype **G** frontal view of male paratype.

**Male** (Figs 5A–C, 6C, D, F, G). Total length 3.03. Carapace 1.70 long, 1.21 wide. Abdomen 1.52 long, 0.98 wide. Clypeus 0.06 high. Eye sizes and inter-distances: AME 0.41, ALE 0.26, PLE 0.23, AERW 0.94, PERW 0.86, EFL 0.64. Legs: I 2.92 (0.89 + 1.11 + 0.51 + 0.41), II 2.35 (0.79 + 0.81 + 0.37 + 0.38), III 2.81 (0.92 + 0.88 + 0.61 + 0.40), IV 3.08 (0.92 + 0.98 + 0.72 + 0.46). Carapace dark brown, cephalic part almost square, thoracic part sloping abruptly, with scattered white setae laterally. Fovea longitudinal, bar shaped. Clypeus dark brown. Chelicerae brown, with two promarginal teeth and one retromarginal enlarged tooth. Endites, labium and sternum colored as chelicerae but paler. Sternum slightly longer than wide, covered with dark setae. Legs yellow to black. Abdomen elongated oval, speckled laterally, with several chevrons posteriorly, covered with white setae; venter yellow-brown with spots.

Palp (Fig. 5A–C): Patella and tibia dark brown, slightly longer than wide, ventral tibial bump stout; RTA slightly shorter than tibia in retrolateral view, tapering to a slightly hooded tip; cymbium dark brown, longer than wide, widest medially, dorsally with a few short, stout spines in the middle; bulb longer than wide, with sperm duct relatively stout, meandering retrolaterally and tapering prolaterally; tegular lobe distinct, covering tibia; embolus with coiled base; embolic terminus flat with small cusps and thin membrane.

**Female** (Fig. 6A, B, E). Total length 3.72. Carapace 1.84 long, 1.36 wide. Abdomen 2.02 long, 1.36 wide. Clypeus 0.07 high. Eye sizes and inter-distances: AME 0.47, ALE 0.28, PLE 0.25, AERW 0.82, PERW 0.77, EFL 0.56. Legs: I 3.00 (0.97 + 1.14 + 0.47 + 0.42), II 2.65 (0.90 + 0.92 + 0.44 + 0.39), III 3.20 (1.10 + 1.02 + 0.66 + 0.42), IV 3.69 (1.10 + 1.19 + 0.89 + 0.51). Habitus similar to that of male except paler.

Epigyne (Fig. 6A, B) slightly wider than long, windows large, separated by median septum; copulatory openings on each side of median septum located posteriorly; copulatory ducts wrap around spermathecae; accessory glands adjacent to copulatory openings; spermathecae spherical; fertilization ducts originating from the median anterior edge of spermathecae, extending almost transversely.

##### Distribution.

Known only from the type locality in Yunnan, China.

#### Genus Foliabitus

Taxon classificationAnimaliaAraneaeSalticidae

Zhang & Maddison, 2012

37DF7257-546F-542E-800A-C34FA7F62A5A

##### Type species.

*Foliabitus longzhou* Zhang & Maddison, 2012.

#### Foliabitus weihangi sp. nov.

Taxon classificationAnimaliaAraneaeSalticidae

386CF993-C96B-5180-A325-C596FD3A4E05

http://zoobank.org/CC5F3D88-678D-4BA7-9591-8A83B0D6CEC6

Figures 7 , 8

##### Type material.

***Holotype*** ♂(IZCAS-Ar40399), China: Yunnan: Xishuangbanna, Mengla County, Menglun Township, XTBG, Leprosy Village, 21.8932N, 101.2883E, elevation ca 550 m, 09.V.2018, Weihang Wang leg. ***Paratypes*** 4♂4♀ (IZCAS-Ar40400–Ar40407), China: Yunnan: Xishuangbanna, Mengla County, Menglun Township, XTBG, Leprosy Village, 21.8986N, 101.2683E, elevation ca 523 m, 29.IV.2019, Zilong Bai leg.

##### Etymology.

The species is named after Mr. Weihang Wang, who has helped us greatly with this research; noun (name) in genitive case.

##### Diagnosis.

*Foliabitus weihangi* sp. nov. resembles *F. scutigerus* (Zabka, 1985) and *F. longzhou* Zhang & Maddison, 2012 by the long and coiled embolus, nearly forming a circle, but differs in the following: the RTA is curved towards the bulb medially in ventral view (vs. the RTA straight in ventral view in *F. scutigerus* and *F. longzhou*); the RTA curved without a small cusp distally (vs. with a small cusp distally in *F. longzhou*), the tegular lobe protrudes from the bulb (vs. indistinct in *F. scutigerus* and *F. longzhou*); in the female, the copulatory ducts are S-shaped (vs. C-shaped in *F. longzhou*).

##### Description.

**Figure 7.**
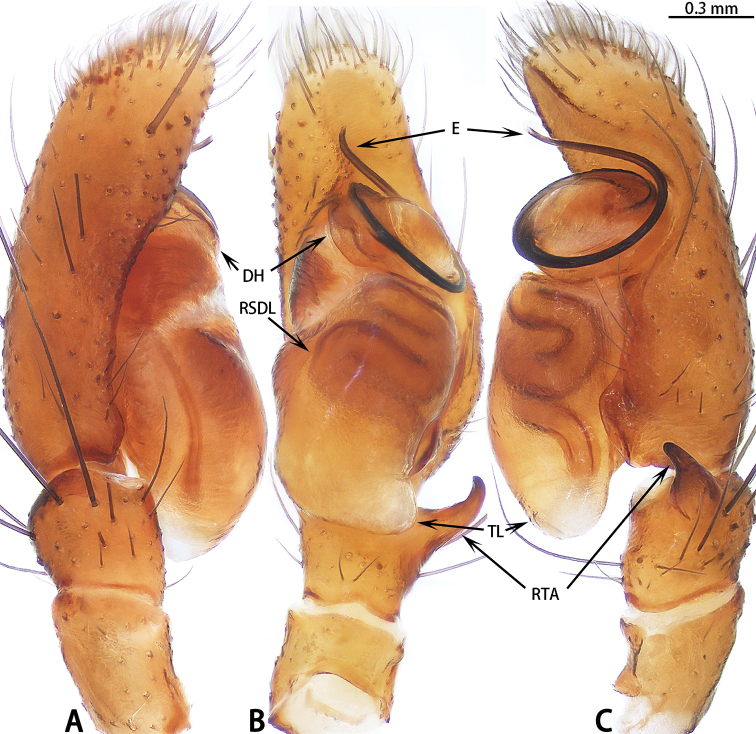
Palp of *Foliabitus weihangi* sp. nov., male holotype. **A** prolateral **B** ventral **C** retrolateral.

**Figure 8.**
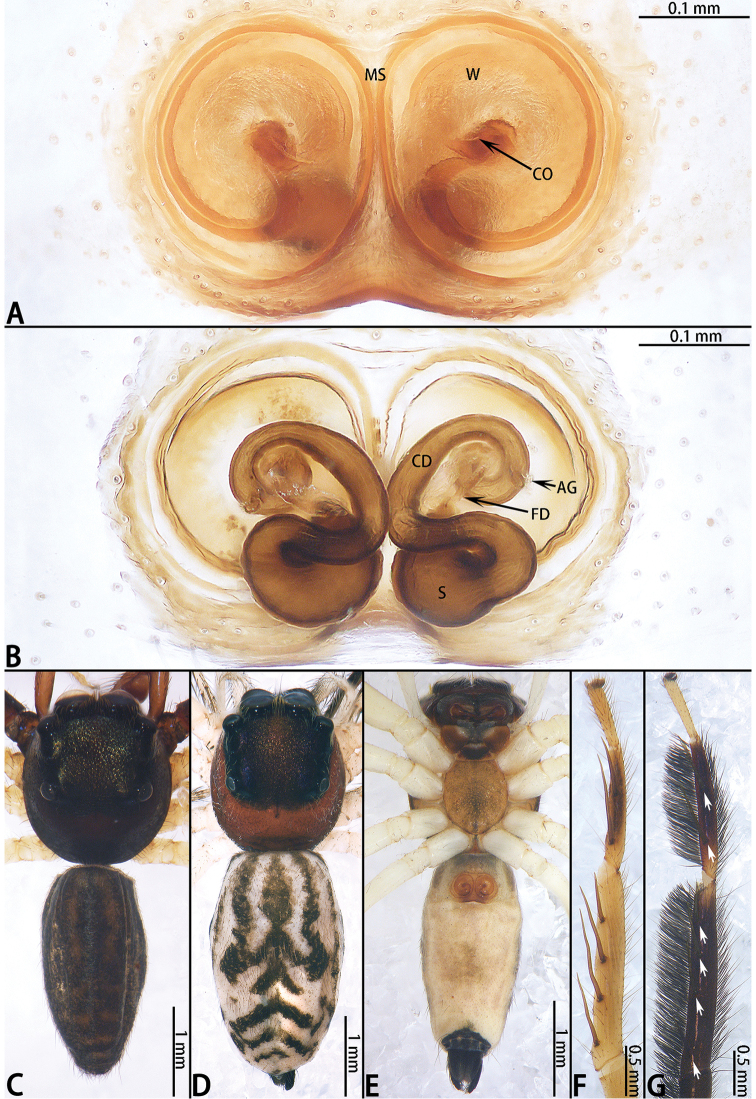
*Foliabitus weihangi* sp. nov., female paratype and male holotype. **A** epigyne, ventral **B** vulva, dorsal **C** male holotype habitus, dorsal **D** female paratype habitus, dorsal **E** same, ventral **F** prolateral view of right leg I, female paratype **G** prolateral view of right leg I, male holotype.

**Male** (Figs 7A–C, 8C, G). Total length 4.29. Carapace 1.94 long, 1.61 wide. Abdomen 2.35 long, 1.18 wide. Clypeus 0.05 high. Eye sizes and inter-distances: AME 0.43, ALE 0.27, PLE 0.25, AERW 1.34, PERW 1.26, EFL 1.02. Legs: I 7.25 (2.13 + 2.88 + 1.47 + 0.76), II 5.14 (1.66 + 1.78 + 1.10 + 0.60), III 5.50 (1.76 + 1.80 + 1.38 + 0.56), IV 5.61 (1.66 + 1.92 + 1.53 + 0.50). Carapace black, cephalic part with dense, green scale-like setae around eyes. Fovea longitudinal, posterior to PLEs. Clypeus yellow, covered with dense, white setae. Chelicerae black, with two retromarginal teeth and one promarginal tooth. Endites and labium dark brown. Sternum brown, covered with dark setae. Legs pale yellow, except leg I black, covered with long, dark setae. Abdomen elongated oval, dorsum black, with pale pattern; venter black with dark setae.

Palp (Fig. 7A–C): Patella red-brown, almost as long as wide; tibia stocky, slightly wider than long, with sclerotized, hook-shaped RTA, curved towards the bulb; cymbium longer than wide, covered with long setae; bulb longer than wide, tegular lobe distinct, curved retrolaterally; embolus long and coiled, nearly forming a circle.

**Female** (Fig. 8A, B, D–F). Total length 4.82. Carapace 2.04 long, 1.63 wide. Abdomen 2.78 long, 1.60 wide. Clypeus 0.06 high. Eye sizes and inter-distances: AME 0.47, ALE 0.28, PLE 0.27, AERW 1.29, PERW 1.26, EFL 1.12. Legs: I 6.16 (1.96 + 2.43 + 1.16 + 0.61), II 5.00 (1.53 + 1.96 + 1.01 + 0.50), III 5.10 (1.59 + 1.66 + 1.31 + 0.54), IV 5.71 (1.66 + 1.98 + 1.53 + 0.54). Habitus (Fig. 8D) similar to that of male except paler. Abdomen dorsally whitish with black pattern similar to male, ventrally pale yellow, with small black triangular patch near spinnerets.

Epigyne (Fig. 8A, B) wider than long, windows large, separated by median septum; copulatory openings at center of windows; copulatory ducts long, S-shaped; spermathecae oval; fertilization ducts well-developed, membranous, lamellar.

##### Distribution.

Known only from the type locality in Yunnan, China.

#### Genus Megaeupoa gen. nov.

Taxon classificationAnimaliaAraneaeSalticidae

1C4A3F2A-DD63-5647-99A7-5FFA6CE3D7A2

http://zoobank.org/1B7801B2-2A3E-454C-9C4D-9083A5864DF1

##### Type species.

*Megaeupoa yanfengi* sp. nov.

##### Etymology.

The generic name is a combination of the word *Mega* and *Eupoa*, referring to the large size and evolutionary relationship of this new genus. The gender is feminine.

##### Diagnosis.

*Megaeupoa* gen. nov. resembles *Brettus* Thorell, 1895 morphologically by the stout RTA, long, undulating embolus, membranous conductor and the epigyne has one tortuous copulatory duct, but it differs in the following: an absence of ventral fringes of long, dense hairs on legs I (Caleb, Acharya and Kumar 2018), RTA stout, slightly longer than wide in lateral view (vs. the RTA is three times longer than wide in *Brettus*), half of the embolus is obscured by the embolic sheath (vs. uncovered in *Brettus*), terminal apophysis present (vs. terminal apophysis absent in *Brettus*); in the female, the vulva has two pairs of spermathecae (vs. one pair of spermathecae in *Brettus*) and the copulatory ducts are curled (vs. copulatory ducts straight in *Brettus*).

##### Description.

**Male.** Total length 4.96–5.64. Carapace red-brown, covered with dense, brown setae, posteriorly with white stripes of setae, cephalic part black or brown. Fovea longitudinal. Clypeus black to brown, covered with several white setae. Chelicerae yellow-brown, with five promarginal and 9–13 retromarginal teeth. Endites pale brown. Labium pale brown, covered with brown setae. Sternum colored as endites, covered with brown setae. Legs brown, with long, white, dense setal ring and black ring pattern. Abdomen elongated oval, dorsum with one pair of stripes of dense white setae, transverse dark brown stripes medially; venter pale brown, covered with setae.

Palpal patella covered with dense, white setae dorsally; tibia slightly wider than long, with ventral apophysis, RTA stout, slightly longer than wide in lateral view; cymbium longer than wide; bulb longer than wide; embolus long, undulate, half of the embolus covered by embolic sheath, other half covered by lateral terminal apophysis; conductor membranous, sheet-shaped, adjacent to embolus; median apophysis small; lateral terminal apophysis whip-like, terminal apophysis distinct, stout.

**Female**. Total length 5.51. Habitus similar to those of male except paler.

Epigyne as long as wide; with posterior hood; windows large, oval; copulatory openings located medially; copulatory ducts curled on either side with two pairs of spermathecae; primary spermathecae small, situated anteriorly, secondary spermathecae large.

##### Composition.

This new genus includes two species: *Megaeupoa yanfengi* sp. nov. and *Megaeupoa gravelyi* (Caleb, 2018), comb. nov.

##### Distribution.

China (Yunnan), India.

#### Megaeupoa yanfengi sp. nov.

Taxon classificationAnimaliaAraneaeSalticidae

177115F4-CF6E-5E6F-B027-93BA56DDDA09

http://zoobank.org/32CB491D-7AD6-4CB1-854B-5A0239E4BBCE

Figures 9 , 10 , 11

##### Type material.

***Holotype*** ♂(IZCAS-Ar40906), China: Yunnan: Xishuangbanna, Mengla County, Menglun Township, XTBG, Leprosy Village, 21.8932N, 101.2883E, elevation ca 550 m, 27.IX.2017, Zhigang Chen, Yunchun Li, Qingyuan Zhao and Jincheng Liu leg. ***Paratypes*** 1♂3♀(IZCAS-Ar40907–Ar40910), same locality as holotype, but 19.IX.2012, Yanfeng Tong leg.

##### Etymology.

The species is named after Mr. Yanfeng Tong, who has helped us greatly with this research; noun (name) in genitive case.

##### Species compared.

*Megaeupoa gravelyi* comb. nov., originally described as *Brettus gravelyi* Caleb in Caleb, Acharya and Kumar (2018).

##### Diagnosis.

The male of *Megaeupoa yanfengi* sp. nov. resembles *M. gravelyi* in having a stout RTA, a long, undulate embolus and a membranous conductor but differs in the following: the RTA terminus is flat in ventral view (vs. subtriangular in *M. gravelyi*), the median apophysis is stout (vs. pointed in *M. gravelyi*), the inferior terminal apophysis is present, the terminal apophysis is semicircular (vs. inferior terminal apophysis absent and terminal apophysis subtriangular in *M. gravelyi*), and the lateral terminal apophysis wraps around the terminal apophysis (vs. next to terminal apophysis in *M. gravelyi*).

##### Description.

**Figure 9.**
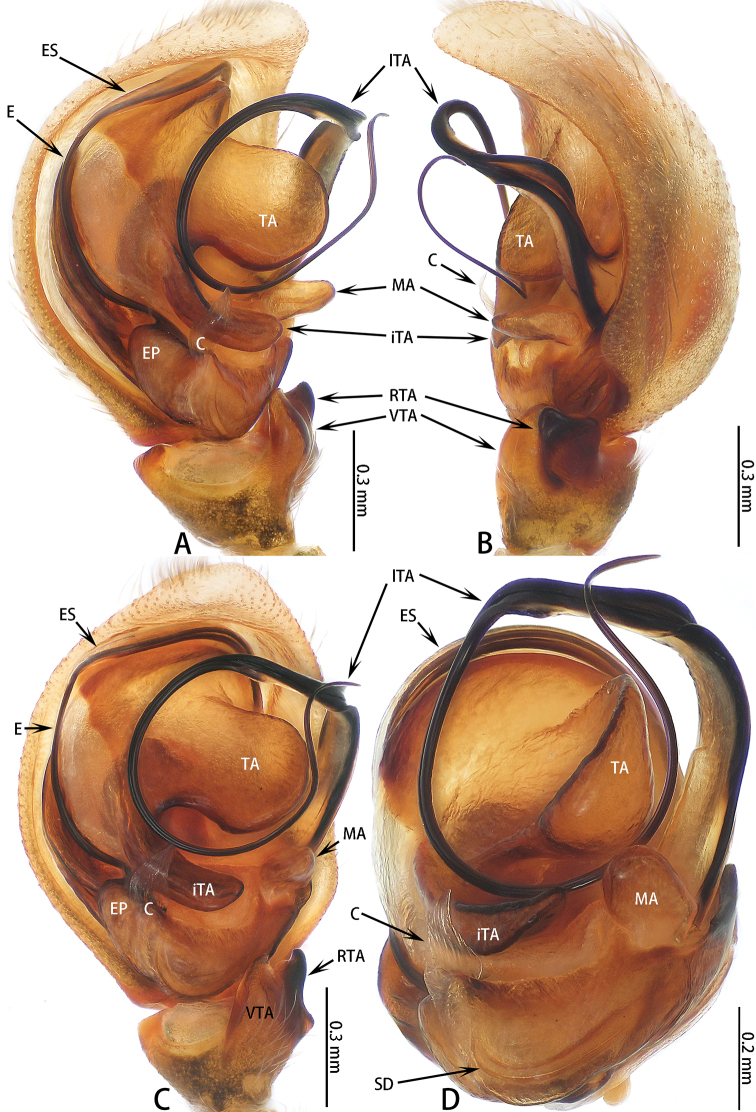
Palp of *Megaeupoa yanfengi* sp. nov. **A–C** male holotype; **D** male paratype. **A** prolateral **B** retrolateral **C** ventral **D** bulb, posterior.

**Figure 10.**
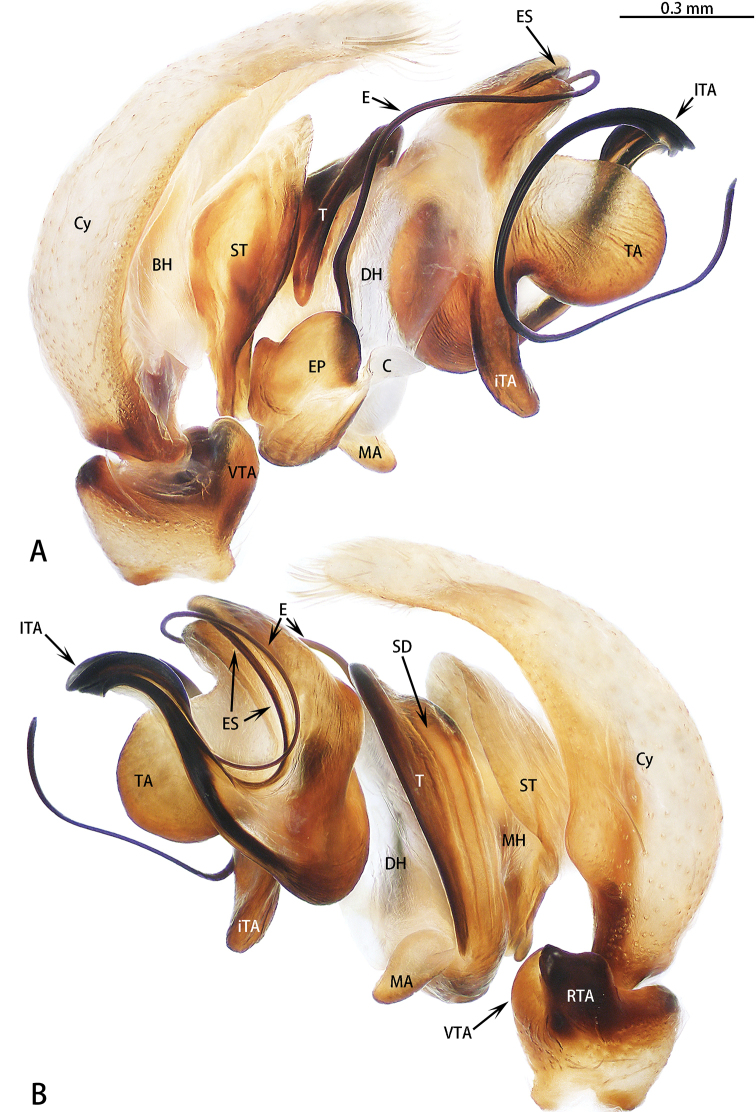
Right palp of *Megaeupoa yanfengi* sp. nov., male paratype (images flipped horizontally). **A** prolateral **B** retrolateral.

**Figure 11.**
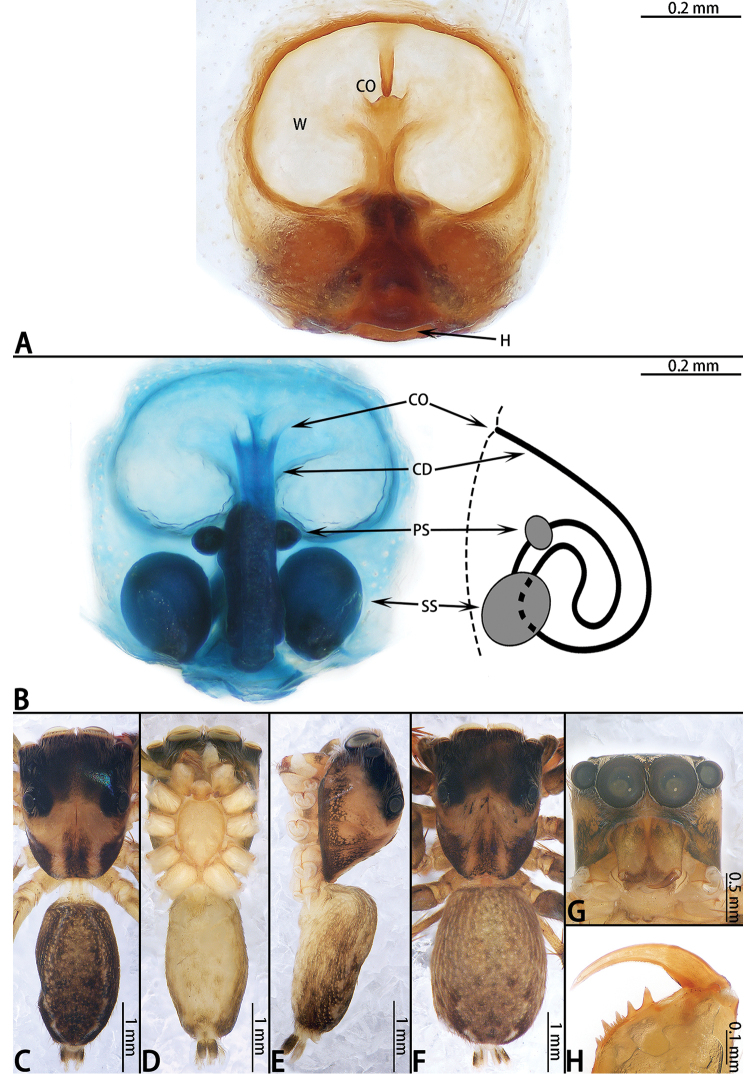
*Megaeupoa yanfengi* sp. nov., female paratype and male holotype. **A** epigyne, ventral **B** vulva, dorsal and schematic duct course shown in lateral view **C** male holotype habitus, lateral **D** male paratype habitus, ventral **E** male paratype habitus, lateral **F** female habitus, dorsal **G** frontal view of male paratype **H** dorsal view of chelicerae, paratype male.

**Male** (Figs 9, 10, 11C–E, G–H). Total length 5.64. Carapace 2.23 long, 1.74 wide. Abdomen 2.94 long, 1.33 wide. Clypeus 0.09 high. Eye sizes and inter-distances: AME 0.59, ALE 0.36, PLE 0.33, AERW 1.64, PERW 1.57, EFL 1.10. Legs: I 4.48 (1.36 + 1.64 + 0.89 + 0.59), II 4.28 (1.30 + 1.53 + 0.87 + 0.58), III 4.48 (1.33 + 1.50 + 1.02 + 0.63), IV 6.12 (1.84 + 1.98 + 1.63 + 0.67). Carapace red-brown, covered with dense, brown setae, posteriorly with white stripes of setae, cephalic part black. Fovea longitudinal. Clypeus black to brown, covered with several white setae. Chelicerae yellow-brown, with five promarginal and nine retromarginal teeth. Endites pale brown. Labium pale brown, covered with brown setae. Sternum colored as endites, covered with brown setae. Legs brown, with long, white, dense setal annulations. Abdomen elongated oval, dorsum with one pair of stripes with dense, white setae, transverse dark brown stripes medially; venter pale brown, covered with setae.

Palp (Figs 9A–D, 10A, B): Patella covered with dense, white setae dorsally; tibia slightly wider than long, with subtriangular ventral apophysis, RTA stout, slightly longer than wide in lateral view, terminus flat; cymbium longer than wide; bulb longer than wide; embolus filiform, undulate, half of embolus obscured by embolic sheath, other half enclosed by lateral terminal apophysis; conductor membranous, sheet-shaped, adjacent to embolus; median apophysis three times longer than wide, stout; inferior terminal apophysis thin, four times longer than wide, lateral terminal apophysis filiform, embolus curled circularly, terminal apophysis semicircular.

**Female** (Fig. 11A, B, F). Total length 5.51. Carapace 2.35 long, 1.76 wide. Abdomen 3.09 long, 1.90 wide. Clypeus 0.09 high. Eye sizes and inter-distances: AME 0.62, ALE 0.37, PLE 0.26, AERW 1.87, PERW 1.71, EFL 1.22. Legs: I 4.29 (1.34 + 1.53 + 0.84 + 0.58), II 4.08 (1.27 + 1.38 + 0.88 + 0.55), III 4.33 (1.23 + 1.48 + 1.01 + 0.61), IV 5.81 (1.59 + 2.01 + 1.50 + 0.71). Habitus similar to that of male.

Epigyne (Fig. 11A, B) as long as wide; hood located posteriorly; windows large, oval; copulatory openings located medially; copulatory ducts curled on either side with two pairs of spermathecae; primary spermathecae small, situated anteriorly, secondary spermathecae larger than primary spermathecae.

##### Distribution.

Known only from the type locality in Yunnan, China.

#### Genus Nigorella

Taxon classificationAnimaliaAraneaeSalticidae

Wesolowska & Tomasiewicz, 2008

476934E8-04B1-51E8-80E8-B777891B20AE

##### Type species.

*Nigorella aethiopic*a Wesolowska & Tomasiewicz, 2008.

#### Nigorella mengla sp. nov.

Taxon classificationAnimaliaAraneaeSalticidae

DF1E3E0B-8447-56C4-8DD1-0305AE8A4564

http://zoobank.org/40E914DD-A047-4855-958A-7DCC3EC0C446

Figures 12 , 13

##### Type material.

***Holotype*** ♂(IZCAS-Ar40911), China: Yunnan: Xishuangbanna, Mengla County, Menglun Township, XTBG, Leprosy Village, 21.8932N, 101.2883E, elevation ca 550 m, 20.IX.2017, Yanfeng Tong leg. ***Paratypes*** 4♂1♀(IZCAS-Ar40912–Ar40916), same data as holotype.

##### Etymology.

The specific name is a noun in apposition and refers to the type locality.

##### Diagnosis.

*Nigorella mengla* sp. nov. resembles *N. sichuanensis* Peng, Xie & Kim, 1993 and *Evarcha orientalis* (Song & Chai, 1992) by the bifurcated RTA and dorsal embolic apophysis behind the embolus but differs in the following: the palpal tibia is wider than long (vs. longer than wide in *N. sichuanensis*); the tegular lobe is folded (vs. straight in *N. sichuanensis* and *E. orientalis*). In the female, the spermathecae are S-shaped (vs. spermathecae coiled in *N. sichuanensis*), and the hoods are deeper (vs. unobvious in *E. orientalis*).

##### Description.

**Figure 12.**
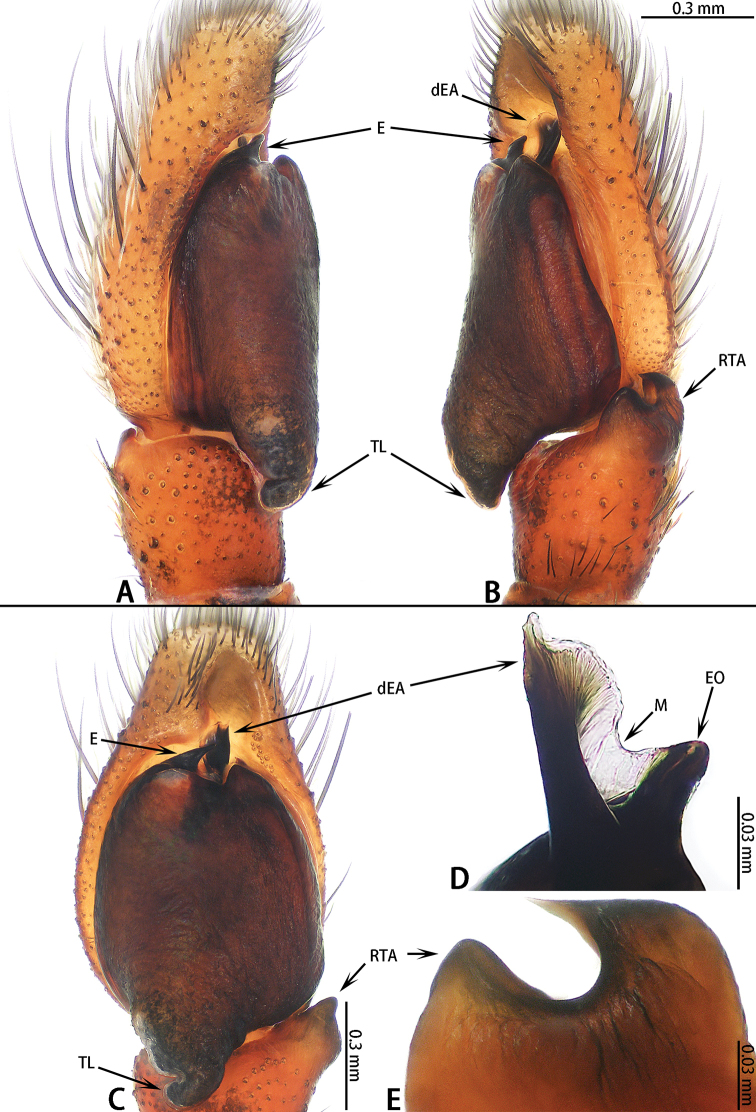
Palp of *Nigorella mengla* sp. nov. **A–C** male holotype; **D, E** male paratype **A** prolateral **B** retrolateral **C** ventral **D** embolic division, dorsal view **E**RTA, retrolateral view.

**Figure 13.**
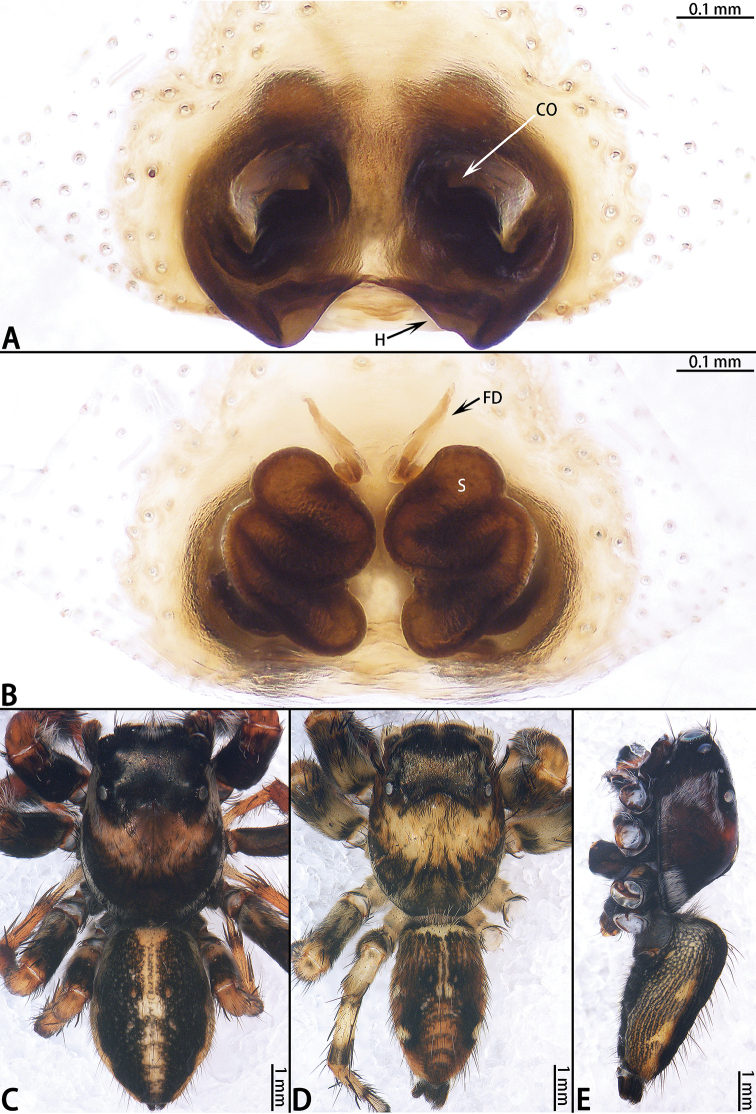
*Nigorella mengla* sp. nov., female paratype and male holotype. **A** epigyne, ventral **B** vulva, dorsal **C** male holotype habitus, dorsal **D** female paratype habitus, dorsal **E** male paratype habitus, lateral.

**Male** (Figs 12, 13C, E). Total length 8.23. Carapace 4.5 long, 3.19 wide. Abdomen 4.04 long, 2.69 wide. Clypeus 0.22 high. Eye sizes and inter-distances: AME 0.79, ALE 0.38, PLE 1.04, AERW 2.35, PERW 2.34, EFL 1.02. Legs: I 7.97 (2.50 + 3.28 + 1.28 + 0.91), II 5.19 (1.64 + 1.88 + 0.96 + 0.71), III 8.01 (2.81 + 2.56 + 1.61 + 1.03), IV 7.85 (2.45 + 2.56 + 1.84 + 1.00). Carapace black, red-brown medially, carapace edge and sides of cephalic part with white setal stripes, thoracic part sloping abruptly, clothed with white and dark setae. Fovea indistinct. Clypeus orange-brown to dark brown, covered with thin setae. Chelicerae black, with two retromarginal teeth and one promarginal tooth. Endites and labium black. Sternum black, covered with dark setae. Legs red-brown except femora with black pattern. Abdomen elongated oval, dorsum with two pairs of muscle depressions, with white line centrally, white line widens medially; venter black with dark setae; sides black with white spots.

Palp (Fig. 12A–E): Tibia slightly wider than long, RTA bifurcated, ventral branch blunt, dorsal ramus well-developed, pointed; cymbium flattened, covered with long setae; bulb almost round, with sperm duct extending along margin, tegular lobe folded; embolus stout, dorsal embolic apophysis behind embolus, connected to embolus with membrane.

**Female** (Fig. 13A, B, D). Total length 7.85. Carapace 4.30 long, 2.94 wide. Abdomen 4.12 long, 2.04 wide. Clypeus 0.19 high. Eye sizes and inter-distances: AME 0.65, ALE 0.41, PLE 0.33, AERW 2.36, PERW 2.35, EFL 1.02. Legs: I 6.43 (2.20 + 2.58 + 0.92 + 0.73), II 6.10 (2.00 + 2.45 + 0.88 + 0.77), III 7.42 (2.56 + 2.50 + 1.48 + 0.88), IV 7.08 (2.18 + 2.48 + 1.56 + 0.86). Habitus similar to that of male except paler.

Epigyne (Fig. 13A, B) wider than long, with pair of hoods near epigastral furrow; copulatory openings situated medially, C-shaped; copulatory ducts indistinct; spermathecae S-shaped; fertilization ducts well-developed.

##### Distribution.

Known only from the type locality in Yunnan, China.

#### Genus Onomastus

Taxon classificationAnimaliaAraneaeSalticidae

Simon, 1900

0A9D753F-4CE7-56E1-B303-AD01EAAE1F9D

##### Type species.

*Onomastus nigricaudus* Simon, 1900.

#### Onomastus chenae sp. nov.

Taxon classificationAnimaliaAraneaeSalticidae

841D13C6-A640-537F-AF02-B65667DF27A8

http://zoobank.org/5D31CB6D-4D53-48E2-80EB-613A6B860611

Figures 14 , 15

##### Type material.

***Holotype*** ♂(IZCAS-Ar40917), China: Yunnan: Xishuangbanna, Mengla County, Menglun Township, XTBG, Leprosy Village, 21.8932N, 101.2883E, elevation ca 550 m, 27.IX.2017, Zhigang Chen, Yunchun Li, Qingyuan Zhao and Jincheng Liu leg. ***Paratypes*** 5♂8♀(IZCAS-Ar40918–Ar40930), same data as holotype.

##### Etymology.

The species is named after Ms. Chen Zeng, who helped us greatly with this research; noun (name) in genitive case.

##### Diagnosis.

Males of *Onomastus chenae* sp. nov. are similar to *O. kanoi* Ono, 1995 by having the same shaped spur, a mesal branch of conductor, and a wide conductor. However, *O. chenae* sp. nov. can be distinguished by having three apophyses on the median apophysis (vs. two in *O. kanoi*); the epigyne is wider than long (vs. longer than wide in *O. kanoi*), and the copulatory opening is located posteriorly (vs. medially in *O. kanoi*).

##### Description.

**Figure 14.**
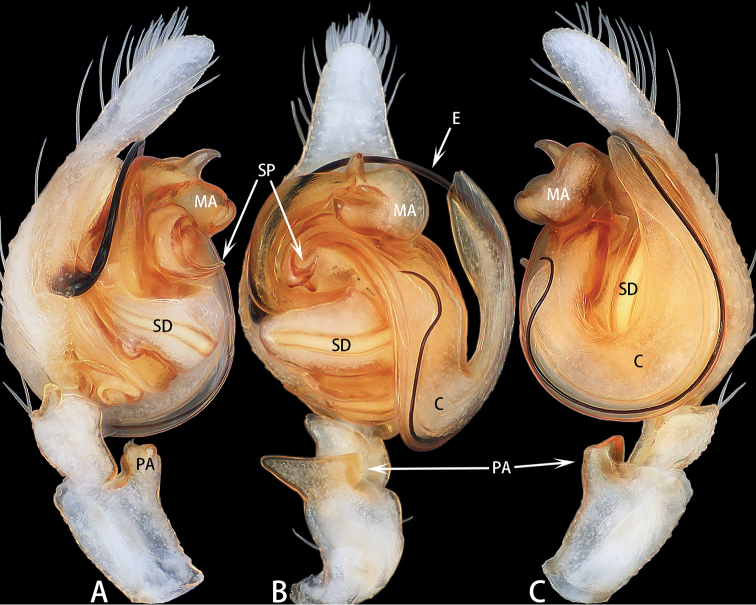
Palp of *Onomastus chenae* sp. nov., male holotype. **A** prolateral **B** ventral **C** retrolateral.

**Figure 15.**
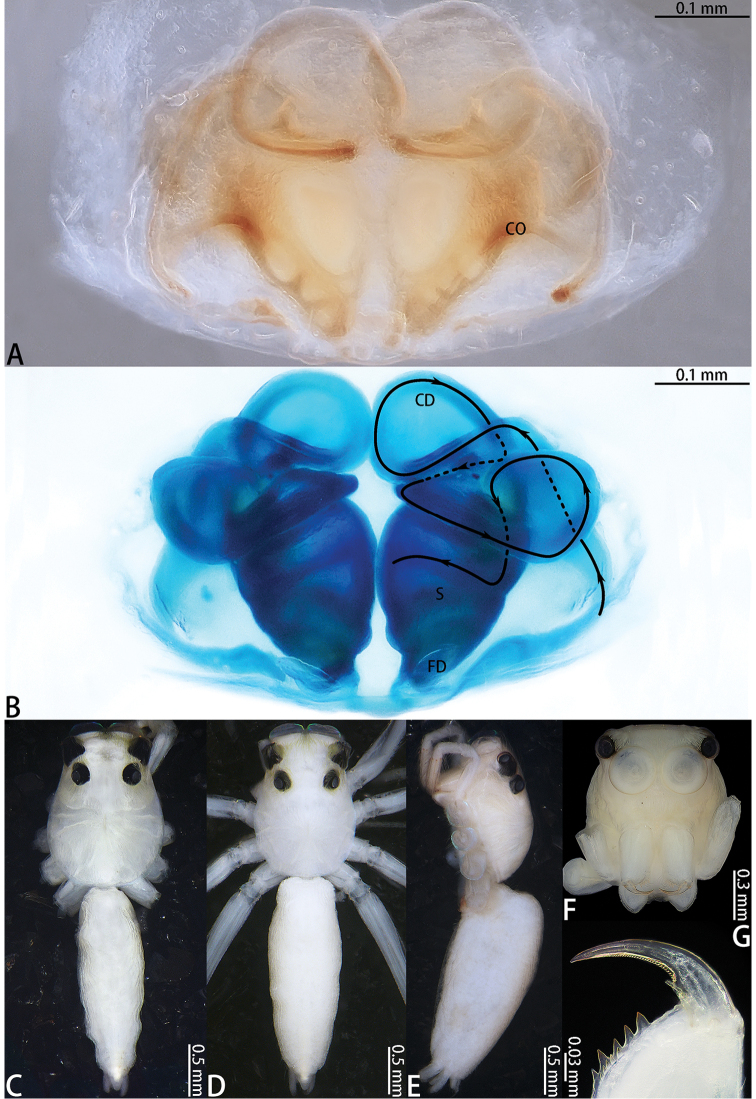
*Onomastus chenae* sp. nov., female paratype and male holotype. **A** epigyne, ventral **B** vulva, dorsal **C** male holotype habitus, dorsal **D** female paratype habitus, dorsal **E** same, lateral **F** frontal view of male paratype **G** dorsal view of chelicerae, paratype.

**Male** (Figs 14A–C, 15C, E–G). Total length 3.66. Carapace 1.60 long, 0.92 wide. Abdomen 2.30 long, 0.75 wide. Clypeus 0.02 high. Eye sizes and inter-distances: AME 0.34, ALE 0.10, PLE 0.12, AERW 0.88, PERW 0.67, EFL 0.47. Legs: I 4.67 (1.28 + 1.85 + 1.00 + 0.44), II 4.55 (1.36 + 1.65 + 1.01 + 0.53), III 4.81 (1.28 + 1.62 + 1.40 + 0.51), IV 5.90 (1.67 + 1.95 + 1.79 + 0.49). Carapace white, black ring around PLEs and PMEs, cephalic part covered with golden setae. Fovea longitudinal, PLEs situated posteriorly. Clypeus white, covered with white setae. Chelicerae white with five promarginal and five retromarginal teeth. Endites, labium and sternum white. Legs white, base of tibia with black spot. Abdomen elongated oval, white.

Palp (Fig. 14A–C): Patella with subtriangular patellar apophysis, longer than wide; tibia as long as wide, without retrolateral apophysis; cymbium longer than wide, covered with setae; bulb approximately as long as wide, structure of bulb is complex; sperm duct clearly visible; spur on mesal branch of conductor hook shaped; conductor wide; embolic division occupying large area on bulb with developed conductor; embolus filiform, very long; median apophysis with three apophyses.

**Female** (Fig. 15A, B, D, E). Total length 3.76. Carapace 1.61 long, 1.05 wide. Abdomen 2.2 long, 0.73 wide. Clypeus 0.02 high. Eye sizes and inter-distances: AME 0.36, ALE 0.10, PLE 0.60, AERW 0.99, PERW 0.70, EFL 1.69. Legs: I 4.59 (1.29 + 1.86 + 1.01 + 0.43), II 4.45 (1.31 + 1.70 + 1.02 + 0.42), III 4.89 (1.31 + 1.59 + 1.45 + 0.54), IV 5.96 (1.68 + 1.98 + 1.76 + 0.54). Habitus similar to that of male.

Epigyne (Fig. 15A, B) wider than long; transverse copulatory openings sclerotized posteriorly; copulatory ducts and spermathecae visible on epigynal surface, copulatory ducts meandering; spermathecae oval posteriorly.

##### Distribution.

Known only from the type locality in Yunnan, China.

#### Genus Synagelides

Taxon classificationAnimaliaAraneaeSalticidae

Strand, 1906

C2F300EE-4446-50DE-A07A-15ADBFDA8762

##### Type species.

*Synagelides agoriformis* Strand, 1906.

#### Synagelides platnicki sp. nov.

Taxon classificationAnimaliaAraneaeSalticidae

EB0862AC-C4DE-5C71-A92E-3E0698BEB9DC

http://zoobank.org/D99DB9E7-6486-4471-9BDD-C5D20635D47B

Figures 16 , 17

##### Type material.

***Holotype*** ♂(IZCAS-Ar40931), China: Yunnan: Xishuangbanna, Mengla County, Menglun Township, XTBG, Leprosy Village, 21.8932N, 101.2883E, elevation ca 550 m, 27.IX.2017, Zhigang Chen, Yunchun Li, Qingyuan Zhao and Jincheng Liu leg. ***Paratypes*** 6♂7♀(IZCAS-Ar40932–Ar40944), same data as holotype.

**Figure 16.**
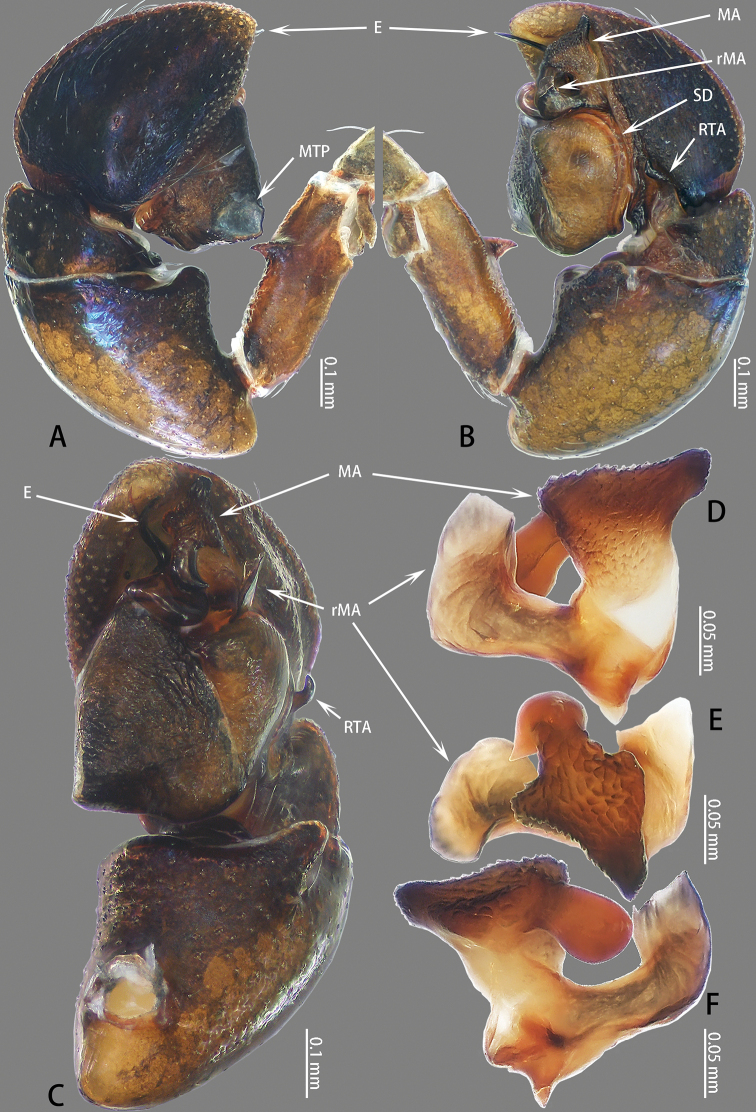
Palp of *Synagelides platnicki* sp. nov. **A–C** male holotype; **D–F** retrolateral median apophysis on right palp, male paratype. **A** prolateral **B** retrolateral **C** ventral **D** retrolateral **E** ventral **F** prolateral.

##### Etymology.

The species is named after the late Norman I. Platnick (1951–2020, see Li 2020b) to commemorate his immense contribution to arachnology; noun (name) in genitive case.

##### Diagnosis.

*Synagelides platnicki* sp. nov. resembles *S. lushanensis* Xie & Yin, 1990 by having the same shaped median apophysis and a coiled embolus but differs by the following: the length of the RTA is four times as long as the length of the cymbium (vs. two times the length in *S. lushanensis*), the dorsal tibial apophysis is absent (vs. present in *S. lushanensis*) and the retrolateral median apophysis is L-shaped in retrolateral view (vs. straight in *S. lushanensis*); in the female, the hood of the epigyne is as long as wide (vs. two times longer than wide in *S. lushanensis*), and the copulatory duct is coiled 360° (vs. S-shaped in *S. lushanensis*).

##### Description.

**Figure 17.**
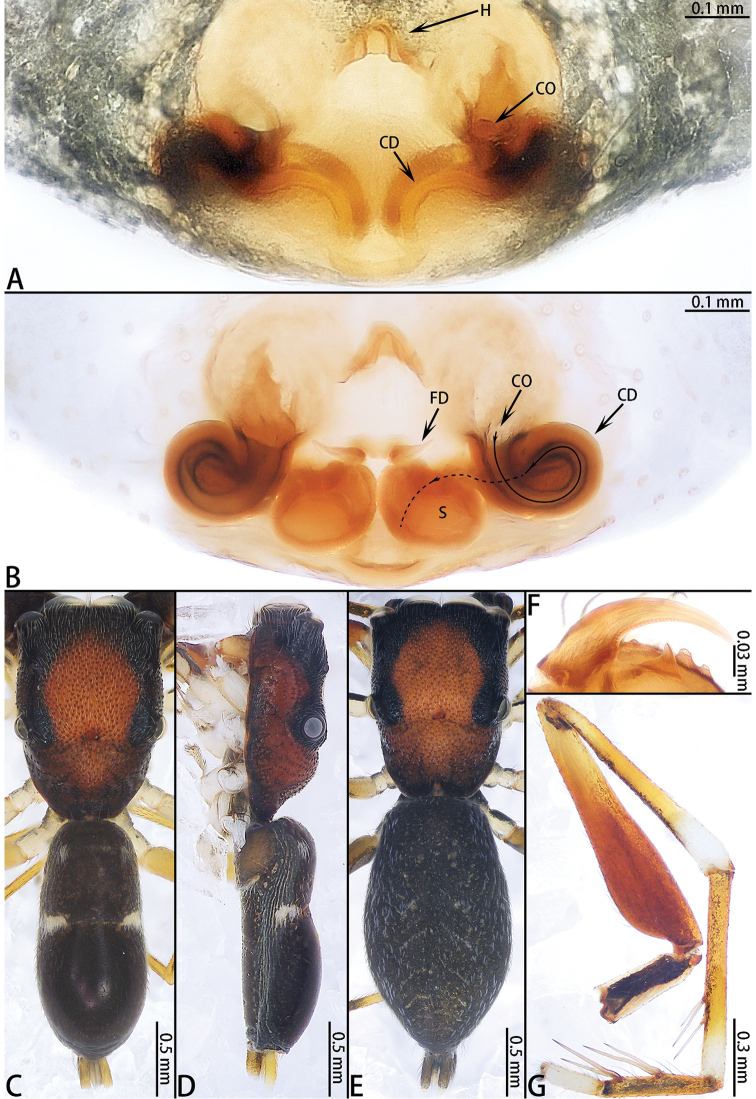
*Synagelides platnicki* sp. nov., female paratype and male holotype. **A** epigyne, ventral **B** vulva, dorsal **C** male holotype habitus, dorsal **D** male paratype habitus, lateral **E** female paratype habitus, dorsal **F** dorsal view of chelicerae, paratype male **G** prolateral view of left leg I, male paratype. female.

**Male** (Figs 16, 17C, D, F, G). Total length 3.22. Carapace 1.50 long, 0.99 wide. Abdomen 1.85 long, 0.75 wide. Clypeus 0.03 high. Eye sizes and inter-distances: AME 0.35, ALE 0.20, PLE 0.19, AERW 0.98, PERW 1.03, EFL 0.90. Legs: I 3.93 (1.18 + 1.02 + 1.00 + 0.44 + 0.29), II 2.14 (0.63 + 0.24 + 0.52 + 0.49 + 0.26), III 2.44 (0.71 + 0.26 + 0.58 + 0.60 + 0.29), IV 3.24 (0.89 + 0.36 + 0.84 + 0.79 + 0.36). Carapace red-brown, widest between coxae II and III, covered with white setae. Clypeus dark brown. Fovea subtriangular. Chelicerae yellow-brown, with two promarginal teeth and one retromarginal tooth. Endites yellow-brown. Sternum brown, covered with thin setae. Femur of leg I red, other femora with black pattern ventrally. Abdomen elongated oval, dorsum with two pairs of white dorso-lateral spots, covered with white setae on the spots and laterally; venter black.

Palp (Fig. 16A–F) femur brown, approximately three times longer than wide, with ventral median apophysis; patella brown, almost as long as wide, with ventral bulge; tibia wider than long, with RTA tapering towards tip, slightly longer than tibia, tip slightly bent ventrally then dorsally; cymbium flattened, widest medially; bulb widest at base; embolus coiled 360°, median apophysis with serrated apophysis, terminus blunt; retrolateral median apophysis L-shaped.

**Female** (Fig. 17A, B, E). Total length 3.68. Carapace 1.48 long, 0.98 wide. Abdomen 2.14 long, 1.03 wide. Clypeus 0.02 high. Eye sizes and inter-distances: AME 0.33, ALE 0.20, PLE 0.20, AERW 1.03, PERW 1.03, EFL 0.85. Legs: I 2.91 (0.88 + 0.69 + 0.70 + 0.37 + 0.27), II 1.99 (0.61 + 0.23 + 0.47 + 0.41 + 0.27), III 2.14 (0.61 + 0.25 + 0.49 + 0.51 + 0.28), IV 2.73 (0.75 + 0.30 + 0.70 + 0.69 + 0.29). Habitus similar to that of male.

Epigyne (Fig. 17A, B) wider than long, with anterior hood, copulatory openings located medially; copulatory ducts visible on epigynal surface, coiled 360°, connecting with anterior edge of spermathecae; spermathecae spherical, touching medially; fertilization ducts originating from the median anterior edge of spermathecae, extending almost transversely.

##### Distribution.

Known only from the type locality in Yunnan, China.

## Discussion

The following two jumping spiders were also collected from Xishuangbanna Tropical Botanical Garden (XTBG).

***Irura mandarina* Simon, 1903**

*Irura mandarina*Simon 1903: 735 (♀); Prószyński 2017: 18, fig. 6D (♀)

*Kinhia prima*Żabka 1985: 233, figs 246–250 (♂) syn. nov.

*Irura prima*: Prószyński 2017: 18, fig. 6E (♂).

**Comments**. Conspecificity of the *Irura mandarina* female and *I. prima* (Żabka, 1985) male is based on a large number of spider specimens collected at the same locality in XTBG with similarities in size and color pattern.

***Ptocasius montiformis* Song, 1991**

*Ptocasius montiformis*Song 1991: 163, figs 1A–D (♀); Song, Zhu and Chen 1999: 543, figs 313T–U (♀)

*Evarcha digitata* Peng & Li 2002: 469, figs 1A–D (♂); Prószyński 2018: 155, fig. 15I (♂) syn. nov.

**Comments**. Conspecificity of the *Ptocasius montiformis* female and *Evarcha digitata* male is based on a large number of spider specimens collected at the same locality in XTBG with similarities in size and color pattern.

Adding the new species reported here, a total of 121 jumping spider species are reported from Xishuangbanna, of which, 77 species (marked with an asterisk) were collected in XTBG by us. A checklist of Xishuangbanna jumping spiders follows, and for a complete list of taxonomic references see WSC (2020).

1. Afraflacilla ballarini Cao & Li, 2016*
2. Agorius tortilis Cao & Li, 2016*
3. Attulus penicillatus (Simon, 1875)*
4. Bavia capistrata (C. L. Koch, 1846)
5. Bavirecta exilis (Cao & Li, 2016)*
6. Bianor angulosus (Karsch, 1879)
7. Bristowia heterospinosa Reimoser, 1934*
8. Burmattus pococki (Thorell, 1895)
9. Burmattus sinicus Prószyński, 1992*
10. Carrhotus sannio (Thorell, 1877)
11. Carrhotus sarahcrewsae Cao & Li, 2016*
12. Carrhotus yunnanensis (Song, 1991)
13. Chalcoscirtus lii Lei & Peng, 2010*
14. Chalcoscirtus nenilini Marusik, 1990*
15. Cheliceroides longipalpis Zabka, 1985*
16. Chinattus dactyloides (Xie, Peng & Kim, 1993)
17. Chinattus wengnanensis Cao & Li, 2016*
18. Chinophrys mengyangensis Cao & Li, 2016*
19. Chrysilla acerosa Wang & Zhang, 2012
20. Cocalus menglaensis Cao & Li, 2016*
21. Colyttus proszynskii Caleb, Chatterjee, Tyagi, Kundu & Kumar, 2018*
22. Colyttus yiwui sp. nov.*
23. Cosmophasis xiaolonghaensis Cao & Li, 2016*
24. Cytaea tongi Wang & Li, 2020*
25. Cytaea yunnanensis Cao & Li, 2016*
26. Dendroicius hotaruae sp. nov.*
27. Dexippus pengi Wang & Li, 2020*
28. Emathis sumatranus Prószyński & Deeleman-Reinhold, 2012*
29. Epeus bicuspidatus (Song, Gu & Chen, 1988)
30. Epeus flavobilineatus (Doleschall, 1859)
31. Epeus indicus Prószyński, 1992
32. Epocilla calcarata (Karsch, 1880)*
33. Euophrys subwanyan Wang & Li, 2020*
34. Euophrys xuyei sp. nov.*
35. Eupoa yunnanensis Peng & Kim, 1997
36. Evarcha orientalis (Song & Chai, 1992)*
37. Evarcha pococki Zabka, 1985
38. Foliabitus weihangi sp. nov.*
39. Gedea fungiformis (Xiao & Yin, 1991)*
40. Gedea pinguis Cao & Li, 2016*
41. Gelotia liuae Wang & Li, 2020*
42. Gelotia syringopalpis Wanless, 1984
43. Gelotia zhengi Cao & Li, 2016*
44. Harmochirus brachiatus (Thorell, 1877)
45. Harmochirus insulanus (Kishida, 1914)*
46. Hasarius adansoni (Audouin, 1826)
47. Hyllus diardi (Walckenaer, 1837)*
48. Icius bamboo Cao & Li, 2016*
49. Icius minimus Wesolowska & Tomasiewicz, 2008*
50. Irura longiochelicera (Peng & Yin, 1991)
51. Irura lvshilinensis Wang & Li, 2020*
52. Irura mandarina Simon, 1903*
53. Irura yunnanensis (Peng & Yin, 1991)*
54. Lechia squamata Zabka, 1985*
55. Megaeupoa yanfengi sp. nov.*
56. Menemerus bivittatus (Dufour, 1831)
57. Myrmapeni borneensis (Peckham & Peckham, 1907)*
58. Myrmaplata plataleoides (O. Pickard-Cambridge, 1869)*
59. Myrmaplata turriformis (Badcock, 1918)*
60. Myrmarachne angusta (Thorell, 1877)
61. Myrmarachne brevis Xiao, 2002
62. Myrmarachne circulus Xiao & Wang, 2004
63. Myrmarachne cornuta Badcock, 1918
64. Myrmarachne elongata Szombathy, 1915*
65. Myrmarachne gisti Fox, 1936
66. Myrmarachne jacksoni Prószyński & Deeleman-Reinhold, 2010*
67. Myrmarachne lugubris (Kulczyński, 1895)
68. Myrmarachne melanocephala MacLeay, 1839
69. Myrmarachne melanotarsa Wesolowska & Salm, 2002*
70. Nannenus menghaiensis Cao & Li, 2016*
71. Nigorella mengla sp. nov.*
72. Onomastus nigrimaculatus Zhang & Li, 2005
73. Onomastus chenae sp. nov.*
74. Pancorius latus Cao & Li, 2016*
75. Pancorius magnus Zabka, 1985*
76. Phaeacius malayensis Wanless, 1981
77. Phintella accentifera (Simon, 1901)*
78. Phintella arcuata Huang, Wang & Peng, 2015
79. Phintella bifurcata Prószyński, 1992*
80. Phintella debilis (Thorell, 1891)*
81. Phintella dives (Simon, 1899)*
82. Phintella lepidus Cao & Li, 2016*
83. Phintella pygmaea (Wesolowska, 1981)*
84. Phintella sancha Cao & Li, 2016*
85. Phintella suavisoides Lei & Peng, 2013
86. Phintella vittata (C. L. Koch, 1846)
87. Phintelloides jesudasi (Caleb & Mathai, 2014)*
88. Phintelloides versicolor (C. L. Koch, 1846)
89. Plexippus petersi (Karsch, 1878)
90. Portia fimbriata (Doleschall, 1859)*
91. Portia labiata (Thorell, 1887)
92. Portia quei Zabka, 1985
93. Ptocasius kinhi Zabka, 1985*
94. Ptocasius montiformis Song, 1991*
95. Ptocasius paraweyersi Cao & Li, 2016*
96. Ptocasius strupifer Simon, 1901*
97. Rhene albigera (C. L. Koch, 1846)
98. Rhene atrata (Karsch, 1881)
99. Rhene flavigera (C. L. Koch, 1846)*
100. Rhene mengla Wang & Li, 2020 *
101. Rhene rubrigera (Thorell, 1887)
102. Rhene setipes Zabka, 1985*
103. Rhene triapophyses Peng, 1995*
104. Siler semiglaucus (Simon, 1901)
105. Siler zhangae Wang & Li, 2020*
106. Spartaeus jaegeri Logunov & Azarkina, 2008
107. Spartaeus spinimanus (Thorell, 1878)*
108. Spartaeus thailandica Wanless, 1984
109. Stenaelurillus fuscus Cao & Li, 2016*
110. Stertinius borneensis Logunov, 2018*
111. Synagelides cavaleriei (Schenkel, 1963)
112. Synagelides platnicki sp. nov.*
113. Synagelides yunnan Song & Zhu, 1998*
114. Telamonia vlijmi Prószyński, 1984
115. Thiania bhamoensis Thorell, 1887
116. Thiania suboppressa Strand, 1907*
117. Thyene bivittata Xie & Peng, 1995
118. Thyene orientalis Zabka, 1985*
119. Thyene triangula Xie & Peng, 1995*
120. Toxeus maxillosus C. L. Koch, 1846*
121. Zeuxippus yunnanensis Peng & Xie, 1995

## Supplementary Material

XML Treatment for Colyttus

XML Treatment for Colyttus yiwui

XML Treatment for Dendroicius

XML Treatment for Dendroicius hotaruae

XML Treatment for Euophrys

XML Treatment for Euophrys xuyei

XML Treatment for Foliabitus

XML Treatment for Foliabitus weihangi

XML Treatment for Megaeupoa

XML Treatment for Megaeupoa yanfengi

XML Treatment for Nigorella

XML Treatment for Nigorella mengla

XML Treatment for Onomastus

XML Treatment for Onomastus chenae

XML Treatment for Synagelides

XML Treatment for Synagelides platnicki
